# Beware of reflectance confocal microscopy artifacts when searching hyphae in acral skin^☆^^☆☆^

**DOI:** 10.1016/j.abd.2019.04.013

**Published:** 2019-12-31

**Authors:** Elisa Cinotti, Jean Luc Perrot, Pietro Rubegni

**Affiliations:** a Department of Medical, Surgical and Neurological Science, Dermatology Section, University of Siena, S. Maria alle Scotte Hospital, Siena, Italy; b Department of Dermatology, University Hospital of Saint-Etienne, Saint-Etienne, France

Dear Editor,

We read with interest the article from Veasey et al.^1^ that reported a case of tinea nigra of the palm where reflectance confocal microscopy (RCM) was used to confirm the clinical diagnosis. Tinea nigra is a pigmented cutaneous superficial mycosis mainly caused by *Hortaea werneckii*. The typical clinical manifestation of this fungus is a single, brown to black asymptomatic macule with an irregular border mainly localized on palms or soles because infection is believed to occur as a result of inoculation from a contamination source such as soil, sewage, wood, or compost subsequent to trauma in the affected areas. Albeit being usually larger and lighter in color, these lesions tend to resemble acral nevi or melanoma, thus leading many clinicians to perform unnecessary biopsies.

RCM is an emerging non-invasive imaging technique that can show hyphae as thin linear and hyper-reflective structures in the stratum corneum in cutaneous superficial mycosis,^2^, ^3^ including tinea nigra^1^, ^4^ and allows to confirm the clinical diagnosis and to avoid conventional mycological examinations and skin biopsies^1^ (Fig. 1). However, hyphae should be differentiated from the contours of normal keratinocytes that can form thin lines and from artifacts. Curiously, stellate hyper-reflective bodies are often visible in acral skin on RCM, possibly corresponding to keratinocytes membranes in a plane not parallel to the microscope tip.^5^ Fig. 2 shows normal acral skin of a healthy person where these artifacts are well visible and are identical to the images presented by Veasey et al.^1^ as hyphae of tinea nigra. The authors stated that hyphae identified by RCM in tinea nigra were tortuous, irregular, and short, different from the morphology of the thin and elongated hyphae of the dermatophytes. However, in our experience and in the other case of tinea nigra reported in the literature, hyphae of *H. werneckii* are elongated and thin on RCM. Moreover, it is also possible to observe that they are septate. The case of Veasey et al.^1^ highlights the difficulty of identifying and describing for the first time what is not known with the new imaging techniques and suggests caution when making the first descriptions in the medical field.

**Figure 1:**
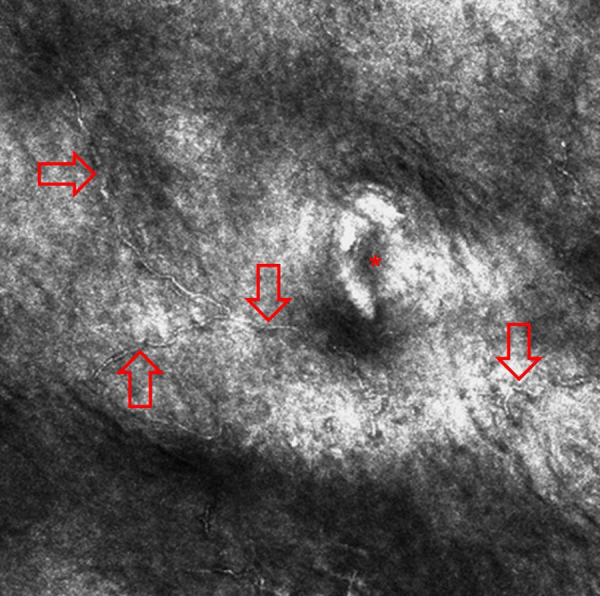
Reflectance confocal microscopy image of a case of tinea nigra reveals thin linear and hyper-reflective structures in upper part of the epidermis (red arrows; the asterisk indicates an acrosyringium).

**Figure 2:**
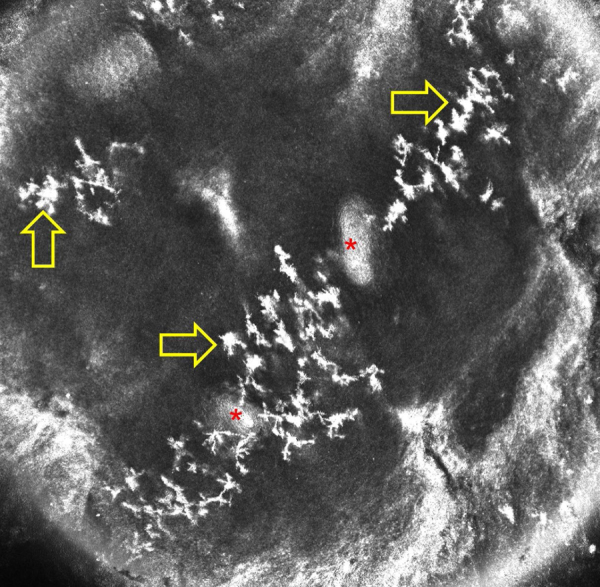
Reflectance confocal microscopy image of normal acral skin of a healthy person shows artifacts presenting as stellate hyper-reflective bodies in the epidermis (yellow arrows; asterisks indicate acrosyringia).

In conclusion, RCM can help to identify hyphae of tinea nigra as for other superficial cutaneous mycosis, but the presence of possible artifacts that may mimic fungal structures in acral skin should be considered for this mycosis that mainly affects palms and soles.

## Financial support

None declared.

## Authors’ contribution

Elisa Cinotti: Approval of the final version of the manuscript; study design and planning; data collection; preparation and writing of the manuscript; critical revision of the manuscript.

Jean Luc Perrot: Approval of the final version of the manuscript; study design and planning; data collection, analysis and interpretation; effective participation in research orientation; intellectual participation in propaedeutic and/or therapeutic conduct of studied cases; critical literature review; critical revision of the manuscript.

Pietro Rubegni: Approval of the final version of the manuscript; effective participation in research orientation; intellectual participation in propaedeutic and/or therapeutic conduct of studied cases; critical revision of the manuscript.

## Conflicts of interest

None declared.
